# Temporal landscape of human gut RNA and DNA virome in SARS-CoV-2 infection and severity

**DOI:** 10.1186/s40168-021-01008-x

**Published:** 2021-04-14

**Authors:** Tao Zuo, Qin Liu, Fen Zhang, Yun Kit Yeoh, Yating Wan, Hui Zhan, Grace C. Y. Lui, Zigui Chen, Amy Y. L. Li, Chun Pan Cheung, Nan Chen, Wenqi Lv, Rita W. Y. Ng, Eugene Y. K. Tso, Kitty S. C. Fung, Veronica Chan, Lowell Ling, Gavin Joynt, David S. C. Hui, Francis K. L. Chan, Paul K. S. Chan, Siew C. Ng

**Affiliations:** 1 Center for Gut Microbiota Research, Faculty of Medicine, The Chinese University of Hong Kong, Shatin, Hong Kong, China; 2 Li Ka Shing Institute of Health Science, The Chinese University of Hong Kong, Shatin, Hong Kong, China; 3 State Key Laboratory for Digestive disease, Institute of Digestive Disease, The Chinese University of Hong Kong, Shatin, Hong Kong, China; 4 Department of Medicine and Therapeutics, Faculty of Medicine, The Chinese University of Hong Kong, Shatin, Hong Kong, China; 5 Microbiota I-Center (MagIC), The Chinese University of Hong Kong, Hong Kong, China; 6 Department of Microbiology, The Chinese University of Hong Kong, Shatin, Hong Kong, China; 7 Stanley Ho Centre for Emerging Infectious Diseases, The Chinese University of Hong Kong, Shatin, Hong Kong, China; 8 Department of Medicine and Geriatrics, United Christian Hospital, Hong Kong, China; 9 Department of Pathology, United Christian Hospital, Hong Kong, China; 10 Department of Anaesthesia and Intensive Care, Faculty of Medicine, The Chinese University of Hong Kong, Shatin, Hong Kong, China

## Abstract

**Background:**

Coronavirus disease 2019 (COVID-19) caused by the enveloped RNA virus SARS-CoV-2 primarily affects the respiratory and gastrointestinal tracts. SARS-CoV-2 was isolated from fecal samples, and active viral replication was reported in human intestinal cells. The human gut also harbors an enormous amount of resident viruses (collectively known as the virome) that play a role in regulating host immunity and disease pathophysiology. Understanding gut virome perturbation that underlies SARS-CoV-2 infection and severity is an unmet need.

**Methods:**

We enrolled 98 COVID-19 patients with varying disease severity (3 asymptomatic, 53 mild, 34 moderate, 5 severe, 3 critical) and 78 non-COVID-19 controls matched for gender and co-morbidities. All subjects had fecal specimens sampled at inclusion. Blood specimens were collected for COVID-19 patients at admission to test for inflammatory markers and white cell counts. Among COVID-19 cases, 37 (38%) patients had serial fecal samples collected 2 to 3 times per week from time of hospitalization until after discharge. Using shotgun metagenomics sequencing, we sequenced and profiled the fecal RNA and DNA virome. We investigated alterations and longitudinal dynamics of the gut virome in association with disease severity and blood parameters.

**Results:**

Patients with COVID-19 showed underrepresentation of Pepper mild mottle virus (RNA virus) and multiple bacteriophage lineages (DNA viruses) and enrichment of environment-derived eukaryotic DNA viruses in fecal samples, compared to non-COVID-19 subjects. Such gut virome alterations persisted up to 30 days after disease resolution. Fecal virome in SARS-CoV-2 infection harbored more stress-, inflammation-, and virulence-associated gene encoding capacities including those pertaining to bacteriophage integration, DNA repair, and metabolism and virulence associated with their bacterial host. Baseline fecal abundance of 10 virus species (1 RNA virus, pepper chlorotic spot virus, and 9 DNA virus species) inversely correlated with disease COVID-19 severity. These viruses inversely correlated with blood levels of pro-inflammatory proteins, white cells, and neutrophils. Among the 10 COVID-19 severity-associated DNA virus species, 4 showed inverse correlation with age; 5 showed persistent lower abundance both during disease course and after disease resolution relative to non-COVID-19 subjects.

**Conclusions:**

Both enteric RNA and DNA virome in COVID-19 patients were different from non-COVID-19 subjects, which persisted after disease resolution of COVID-19. Gut virome may calibrate host immunity and regulate severity to SARS-CoV-2 infection. Our observation that gut viruses inversely correlated with both severity of COVID-19 and host age may partly explain that older subjects are prone to severe and worse COVID-19 outcomes. Altogether, our data highlight the importance of human gut virome in severity and potentially therapeutics of COVID-19.

**Video Abstract.**

**Supplementary Information:**

The online version contains supplementary material available at 10.1186/s40168-021-01008-x.

## Introduction

A novel RNA virus, SARS-CoV-2, has caused a global pandemic of coronavirus disease 2019 (COVID-19) since its emergence in December 2019. Although most cases of COVID-19 are mild, disease severity varies substantially across patients, and severe cases can result in respiratory failure or death [1]. A significant amount of patients with COVID-19 suffered gastrointestinal (GI) symptoms such as vomiting and diarrhea [2–5]. In addition, SARS-CoV-2 viral RNA and transcriptional activity were detected in fecal samples [6–8], suggesting that the GI tract is an extra-pulmonary site for virus replication and activity.

The GI tract harbors the most abundant viruses in the human body collectively known as the gut virome [9, 10]. It encompasses both RNA and DNA viruses that chronically infect their eukaryotic (humans, animals, plants) and prokaryotic hosts (bacteria, amoeba) during a steady state [9]. The gut viral communities *en masse* modulate both the innate and adaptive immunity of the host and the ecology of the host gut microbiota [10]. This “normal” immune state varies from person to person and changes over time. Substantial variation in human immune responses is largely driven by both environmental influences [11] and infection by pathogens [9, 12]. Sequential infection or colonization with acute and chronic viruses can alter host hemostatic immune gene expression and affect response to vaccine [12]. The viral carrier state and its association with host immune-phenotype are under-studied due to a lack of appropriate tools to detect and quantify extremely diverse members of the virome. With the advancement of high-throughput sequencing technology, the highly individual-specific human viromes consisting of members causing acute disease or chronically colonized in asymptomatic individuals are increasingly being uncovered [13, 14]. We have previously demonstrated that the gut microbiome was significantly perturbed in COVID-19 and associated with disease severity and symptoms [8, 15, 16]. Given the co-residing and co-evolutionary nature of the viral/phage and bacterial communities in human gut, we herein hypothesize that the gut virome is altered in patients with COVID-19 and that subject’s baseline virome configurations may associate with immune defense and therefore severity to SARS-CoV-2 infection. We employed RNA and DNA metagenomics to profile the enormous amount of viral members in fecal samples and correlated that with disease immune-phenotype to unravel host-viral relationship and potentially mechanisms underlying severity and recovery in COVID-19.

## Results

Here, we enrolled 98 hospitalized patients with COVID-19 (mean age 33 years; 53% male) and 78 non-COVID-19 controls matched for gender and co-morbidities (mean age 48 years; 42% male, Table 1). All COVID-19 patients and non-COVID-19 controls had stool specimens sampled at inclusion. Blood specimens were additionally sampled for COVID-19 patients at admission to test for pro-inflammatory markers and white cell counts (Supplementary Table 1). Among the COVID-19 patients, 37 (38%) had serial fecal samples collected from hospitalization until after discharge (Supplementary Figure 1). We enriched both fecal RNA and DNA virions from a total of 277 fecal samples and performed non-targeted shotgun metagenomic sequencing on the RNA virome (mostly eukaryotic viruses) and DNA virome (mostly prokaryotic bacteriophages). We report gut virome profiles in association with SARS-CoV-2 infection, disease severity, and blood parameters.

**Table 1:** Clinical characteristics of COVID-19 patients and non-COVID-19 controls

Variables	COVID-19 cases	Non-COVID-19 controls
**Number**	98	78
**Male**	52 (53%)	33 (42%)
**Age, years (mean ± s.e.)**	37 (± 2)	45 (±2)
**Co-morbidities**	55 (56%)	24 (31%)
**Symptoms at admission**		
Fever	38 (38%)	
**Gastrointestinal symptoms**		
Diarrhea	17 (17%)	
**Respiratory symptoms**		
Cough	40 (40%)	
Sputum	18 (18%)	
Rhinorrhea	19 (19%)	
Shortness of breath	9 (9%)	
**Blood result**		
Lymphocyte counts (x10^9^/L, normal range 1.1–2.9, median (IQR))	1.2 (1.0, 1.7)	
**Death**	0 (0%)	

### Alterations in fecal RNA virome of COVID-19 patients

**Fig. 1:**
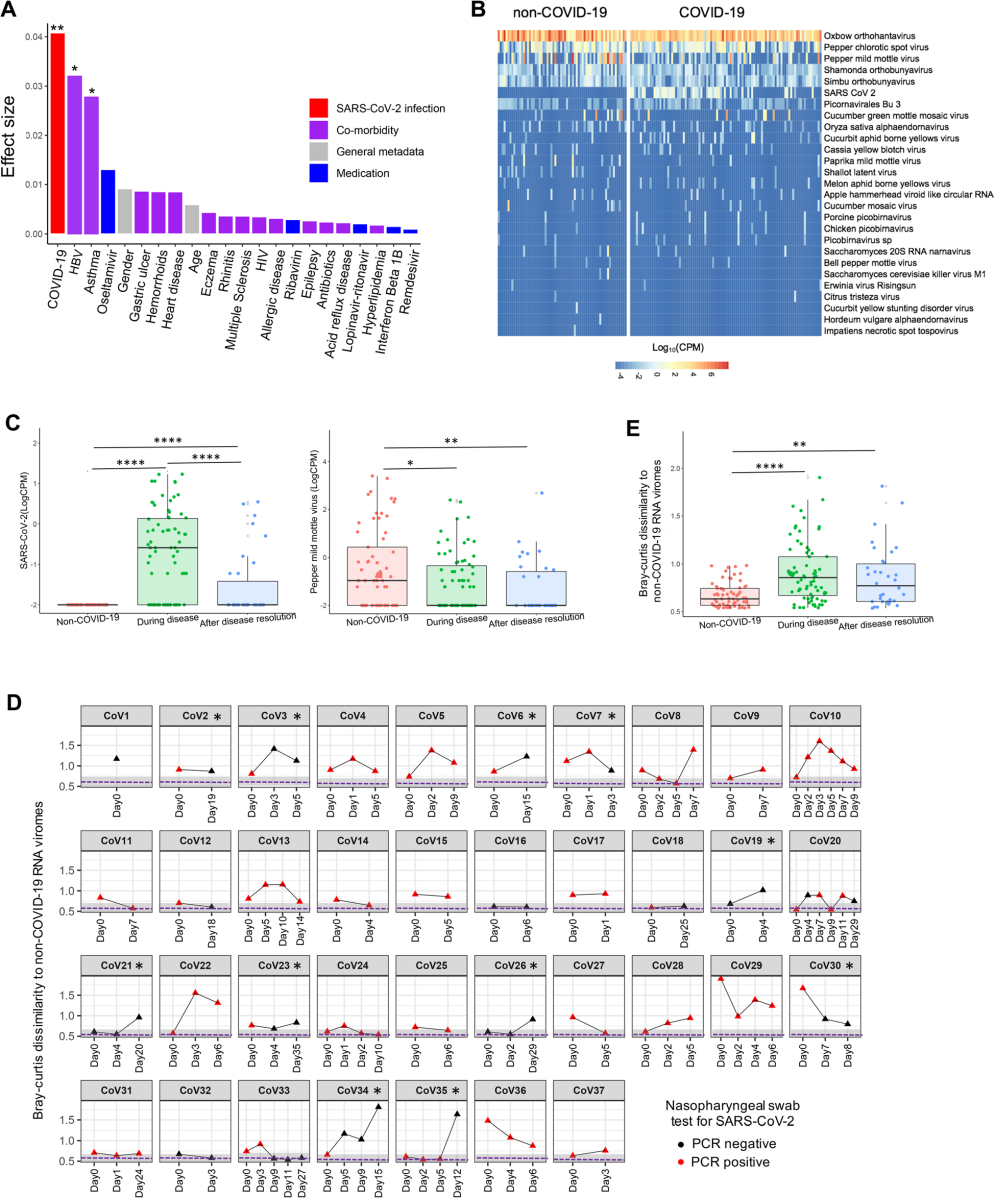
Gut RNA virome in COVID-19 patients and its temporal changes during disease course. **a** Effect size of SARS-CoV-2 infection (COVID-19) and host factors on fecal RNA virome composition variation. The effect size and statistical significance was determined via PERMANOVA analysis with permutation test (*n*=999), **p<0.01, *p<0.05. **b** Heatmap abundance of fecal RNA virus species in COVID-19 patients and non-COVID-19 controls. CPM, count per million reads. **c** The abundance of SARS-CoV-2 and PMMoV in non-COVID-19 controls and in COVID-19 patients during hospitalization and after disease resolution. Between group comparison was conducted by Mann-Whitney test, ****p<0.0001, **p<0.01, *p<0.05. **d** Temporal dissimilarity of patient’s fecal RNA virome to non-COVID-19 fecal RNA viromes over the disease course. Virome dissimilarity of the patient to non-COVID-19 subjects was plotted as the average Bray-Curtis dissimilarity between the indicated fecal virome to all non-COVID-19 viromes. The gray area depicts the dissimilarity range (mean ± s.e.) between non-COVID-19 fecal RNA viromes (the dashed line denotes the mean dissimilarity). “CoV n” denotes COVID-19 patient number. “Day0” denotes baseline date when the first stool was collected after hospitalization; the following time points starting with “Day” represents days since baseline stool collection. Patients labeled with asterisk were those who had markedly more dissimilar DNA virome to non-COVID-19 controls after disease resolution versus their baseline differences to non-COVID-19 controls. **e** The average (median) Bray-Curtis dissimilarity of patient fecal RNA virome to non-COVID-19 fecal RNA virome during the disease course. For box plots, the boxes extend from the 1st to 3rd quartile (25th to 75th percentile), with the median depicted by a horizontal line. Statistical significance was determined by Mann-Whitney test, ****p<0.0001, **p<0.01

To understand whether SARS-CoV-2 infection influences the gut RNA virome, we compared fecal RNA virome of COVID-19 patients at baseline (day 0, the first time point of stool collection after hospitalization) with that of non-COVID-19 controls. Among all host factors (SARS-CoV-2 infection, age, gender, medications, co-morbidities), SARS-CoV-2 infection showed the largest effect size on impacting composition of the fecal RNA virome (permanova test *p*<0.01, *R*^2^=0.041, Fig. 1a) followed by chronic hepatitis B (HBV) infection and asthma. At the species level, SARS-CoV-2 was enriched in fecal samples of patients with COVID-19 compared with non-COVID-19 controls (MaAsLin2 analysis with adjustment for HBV infection and asthma, FDR *p*<0.05, Fig. 1b). In contrast, pepper mild mottle virus (PMMoV), a plant virus known to be prevalent and abundant in human feces [17], was underrepresented in patients with COVID-19 (FDR *p*<0.05, Fig. 1b, c). Seven (19%) of the 37 COVID-19 patients who had longitudinal follow-up showed prolonged fecal SARS-CoV-2 shedding, as indicated by continued SARS-CoV-2 RNA detection in feces after nasopharyngeal clearance of the virus (Supplementary Figure 2A). PMMoV virus was persistently underrepresented both during hospitalization and after disease resolution in COVID-19 patients (Fig. 1c, Supplementary Figure 2B). Diet over the course of hospitalization (Supplementary Table 2) did not show significant effect in the temporal variation of the gut RNA virome (permanova test, *p*=0.2) or the relative abundance of PMMoV virus (permanova test, *p*=0.4).

Overall, the fecal RNA virome composition of COVID-19 patients remained distinct from that of non-COVID-19 controls during the disease course and after disease resolution (Fig. 1d, e). Among 16 COVID-19 patients who had nasopharyngeal clearance of SARS-CoV-2 virus (disease resolution as determined by negative PCR result for SARS-CoV-2 on nasopharyngeal swabs), 11 (69%) had persistently altered fecal RNA virome after disease resolution and two (13%) lasted up to 30 days (Fig. 1d).

We also performed quantitative RT-PCR assays to examine SARS-CoV-2 viral levels in fecal and nasopharyngeal swab specimens in patients with COVID-19 at hospitalization. We found that SARS-CoV-2 levels were lower in feces of patients with moderate COVID-19 than those with asymptotic/mild COVID-19 (*p*<0.01, Supplementary Figure 3A), which was also confirmed by RNA virome shotgun metagenomic sequencing assay (*p*<0.05, Supplementary Figure 3B), though the detection sensitivity of fecal SARS-CoV-2 RNA by shotgun sequencing assay was not on par with quantitative RT-PCR assay. SARS-CoV-2 viral load in fecal specimens was approximately 2-log lower than that in nasopharyngeal specimens (*p*<0.0001, Supplementary Figure 3C). Importantly, SARS-CoV-2 levels in nasopharyngeal samples significantly correlated with SARS-CoV-2 levels in fecal samples (Pearson correlation rho=0.3, *p*=0.0038, Supplementary Figure 3D).

### Alterations in fecal DNA virome of COVID-19 patients

**Fig. 2:**
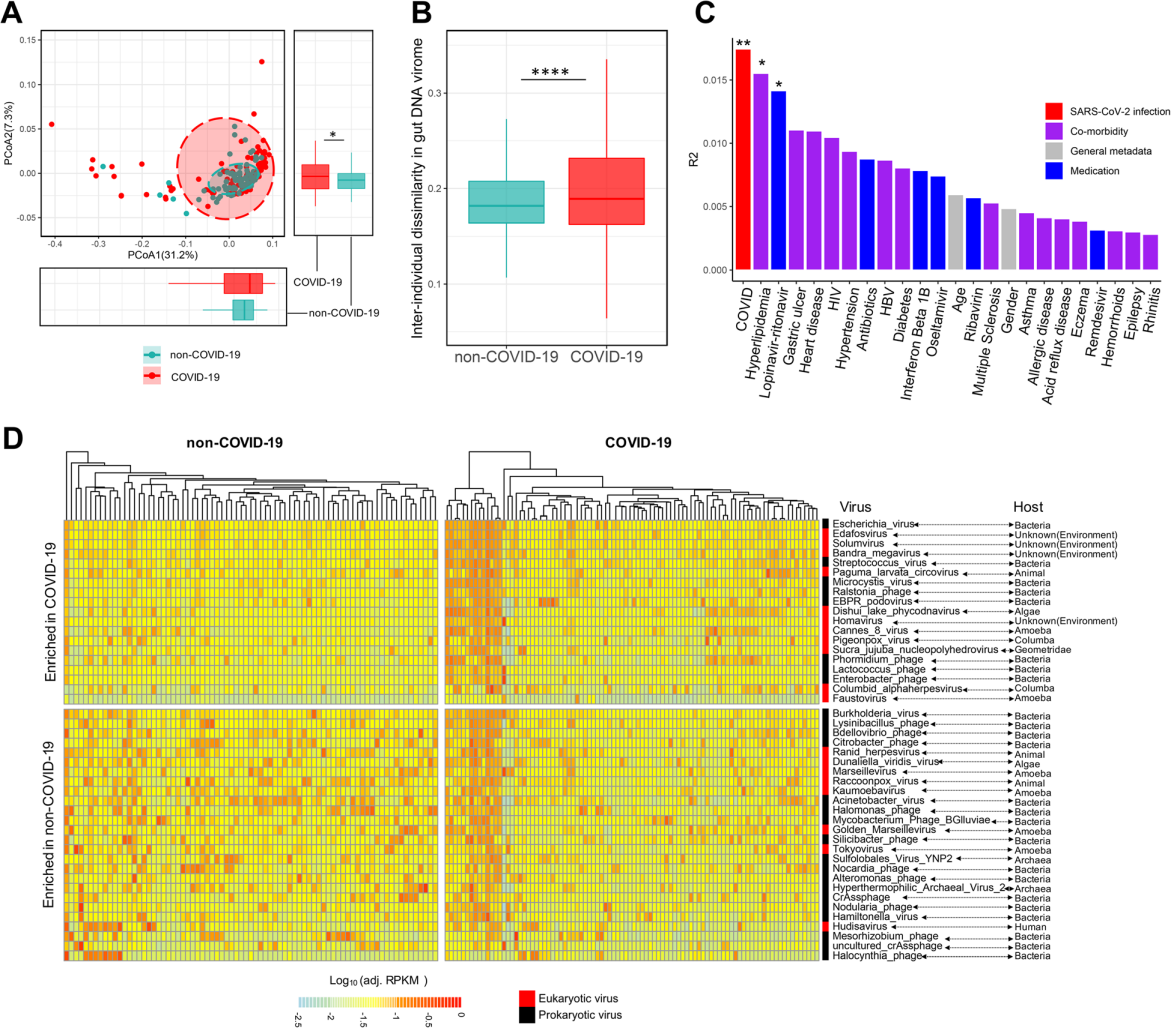
Gut DNA virome alterations in COVID-19 patients. **a** PCoA analysis of fecal DNA viromes in COVID-19 patients versus non-COVID-19 controls. Distribution of fecal viromes along each axis between the two groups was statistically determined by Mann-Whitney test, *p<0.05. **b** Inter-individual dissimilarity (beta-diversity) of fecal DNA viromes within each group. Between-group comparison was conducted by Mann-Whitney test, ****p<0.0001. For box plots, the boxes extend from the 1st to 3rd quartile (25th to 75th percentile), with the median depicted by a horizontal line. **c** Effect size of SARS-CoV-2 infection (COVID-19) and host factors on fecal DNA virome composition variation. The effect size and statistical significance was determined via PERMANOVA analysis with permutation test (*n*=999), **p<0.01, *p<0.05. **d** Differential DNA viruses between COVID-19 patients and non-COVID-19 controls, identified by DESeq, adjusted for antivirals and co-morbidities. Only the significant species (FDR *p*<0.05) were plotted

We then investigated the effect of SARS-CoV-2 infection on fecal DNA virome composition at baseline and during disease course. At the community level, viromes of COVID-19 patients at baseline differed significantly from that of non-COVID-19 controls (permanova *p*<0.01, Fig. 2a) and were more heterogeneous than that of non-COVID-19 controls (*p*<0.0001, Fig. 2b). Among all host factors (SARS-CoV-2 infection, age, gender, medications, co-morbidities), SARS-CoV-2 infection again showed the largest effect size on impacting the composition of the fecal DNA virome (*R*^2^=0.018, Fig. 2c) followed by hyperlipidemia and the antiviral medication Lopinavir-ritonavir. Administration of Lopinavir-ritonavir was inversely associated with the presence of Listeria phage (correlation coefficient −0.21, *p*=0.03), a phage infecting the pathogenic bacteria, Listeria.

A total of 45 DNA virus species were found to be significantly different in the fecal DNA virome between COVID-19 patients and non-COVID-19 controls (19 virus species enriched in COVID-19 patients versus 26 virus species enriched in non-COVID-19 controls, identified via DESeq, while controlling for the factors hyperlipidemia and Lopinavir-ritonavir, shown in Fig. 2d). A majority (69%, 18 out of 26 virus species) of the DNA viruses enriched in feces of non-COVID-19 controls were prokaryotic viruses, particularly bacteriophages (62%, 16 out of 26). In contrast, more eukaryotic viruses, particularly environment-derived eukaryotic viruses with unknown host, were enriched in feces of COVID-19 patients.

**Fig. 3:**
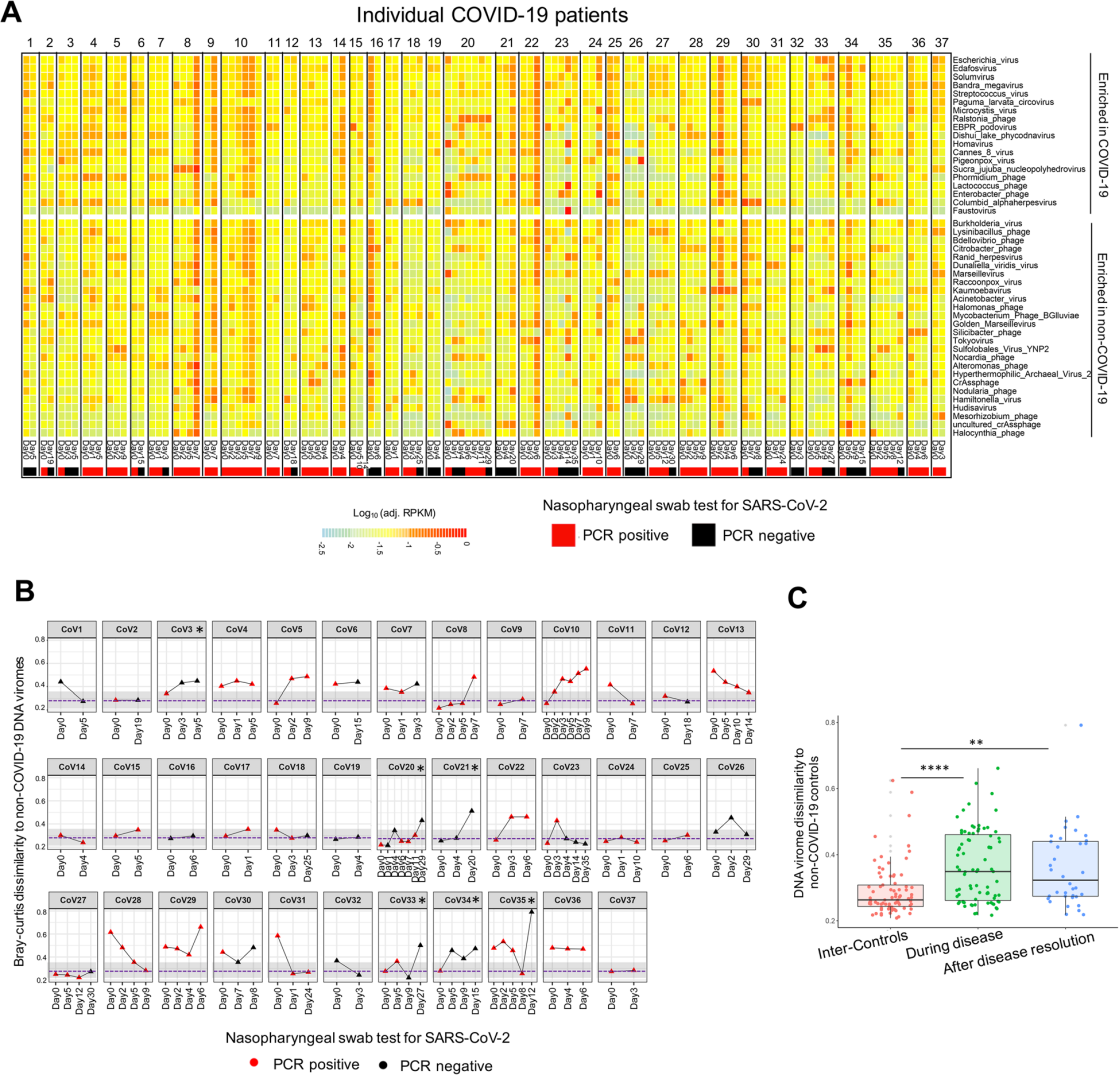
Temporal changes in the gut DNA virome in COVID-19 patients during disease course. **a** Temporal changes of the differential species shown in Fig. 2d in each COVID-19 patient over the disease course. **b** Temporal dissimilarity of patient’s fecal DNA virome to non-COVID-19 fecal DNA viromes over the disease course. Virome dissimilarity of the patient to non-COVID-19 subjects was plotted as the average Bray-Curtis dissimilarity between the indicated fecal virome to all non-COVID-19 viromes. The gray area depicts the dissimilarity range (mean ± s.e.) between non-COVID-19 fecal DNA viromes (the dashed line denotes the mean dissimilarity). “CoV n” denotes COVID-19 patient number. “Day0” denotes baseline date when the first stool was collected after hospitalization; the following time points starting with “Day” represents days since baseline stool collection. Patients labeled with an asterisk were patients who had persisted altered virome after disease resolution. **c** The average (median) Bray-Curtis dissimilarity of patient fecal DNA viromes to non-COVID-19 fecal DNA viromes during the disease course. For box plots, the boxes extend from the 1st to 3rd quartile (25th to 75th percentile), with the median depicted by a horizontal line. Statistical significance was determined by Mann-Whitney test, ****p<0.0001, **p<0.01

The differentially enriched gut DNA virus species in COVID-19 patients showed substantial temporal variations during the disease course (Fig. 3a). Diet during the time of hospitalization did not show significant effect in the temporal variation of the fecal DNA virome (permanova test, *p*=0.3). Overall, fecal DNA virome composition of COVID-19 patients differed markedly from that of non-COVID-19 controls during the disease course and after clearance of SARS-CoV-2 (Fig. 3b, c). Among COVID-19 patients who had follow-up after disease resolution, six (32%) showed markedly more dissimilar fecal DNA virome to non-COVID-19 controls at the last follow-up (three patients lasted up to 20–30 days), compared to their dissimilarity to non-COVID-19 controls at baseline (Fig. 3b).

### Alterations in the functionality of the enteric virome in COVID-19 patients

**Fig. 4:**
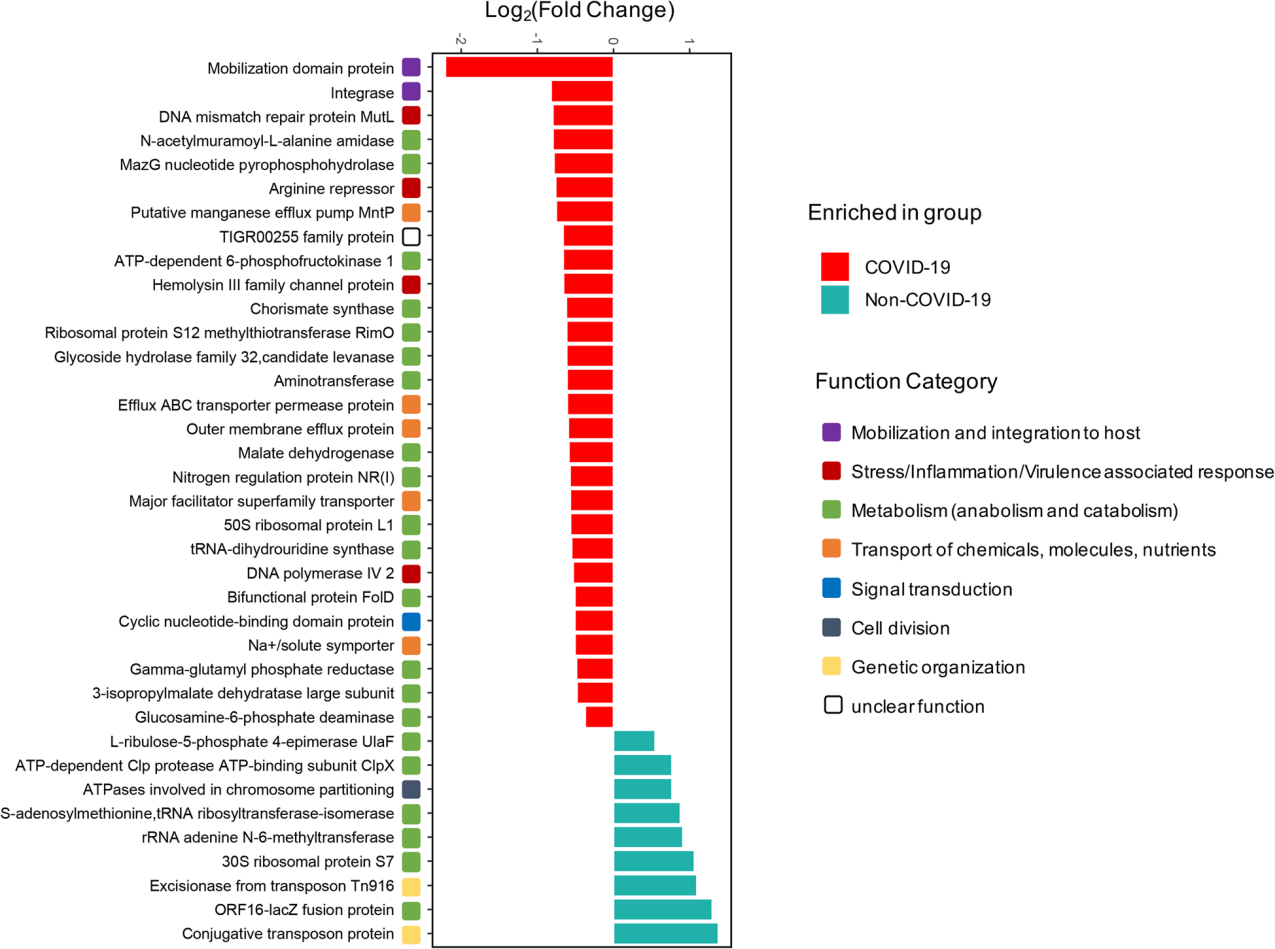
Disparity in the functional capacity of gut virome between COVID-19 patients and non-COVID-19 controls. The differential functions (gene families) were identified by DESeq. Only the significant function terms with FDR *p* value <0.05 and abundance (RPK) >10 were shown

We next investigated functionality alterations of the gut virome using HUMAnN2 prediction. A larger number of gene families were enriched in COVID-19 viromes at baseline than non-COVID-19 controls (28 versus 9 gene families, FDR *p*<0.05, Fig. 4). We found a significant enhancement in the functional capacities of gene mobilization and viral/phage integration into the host in COVID-19 viromes (Fig. 4). Features of viral integration (expansion of temperate virions/phages) have been observed in the gut under inflammatory conditions in both humans and mice [18, 19]. In addition, functions involved in host stress/inflammation/virulence response (DNA repair, Arginine repressor, Hemolysin channel protein, DNA polymerase IV), bacterial metabolism, and membrane transport were also enriched in the fecal virome of COVID-19 patients (Fig. 4). Diet during time of hospitalization did not show significant effect on virome functionality variation (*p*=0.45). These data highlight that SARS-CoV-2 infection may associate with a functionality shift of the human gut virome to inflammation- and stress-related responses in relation to their hosts (both the commensal bacteria and humans). The viral functions enriched in COVID-19 (particularly those associated with host metabolism) were significantly associated with the abundances of viruses enriched in COVID-19, including Streptococcus phage, Escherichia phage, Homavirus, Lactococcus phage, Ralstonia phage, Solumvirus, and Microcystis phage (Supplementary Figure 4).

### Fecal virome alterations correlated with disease severity of COVID-19

**Fig. 5:**
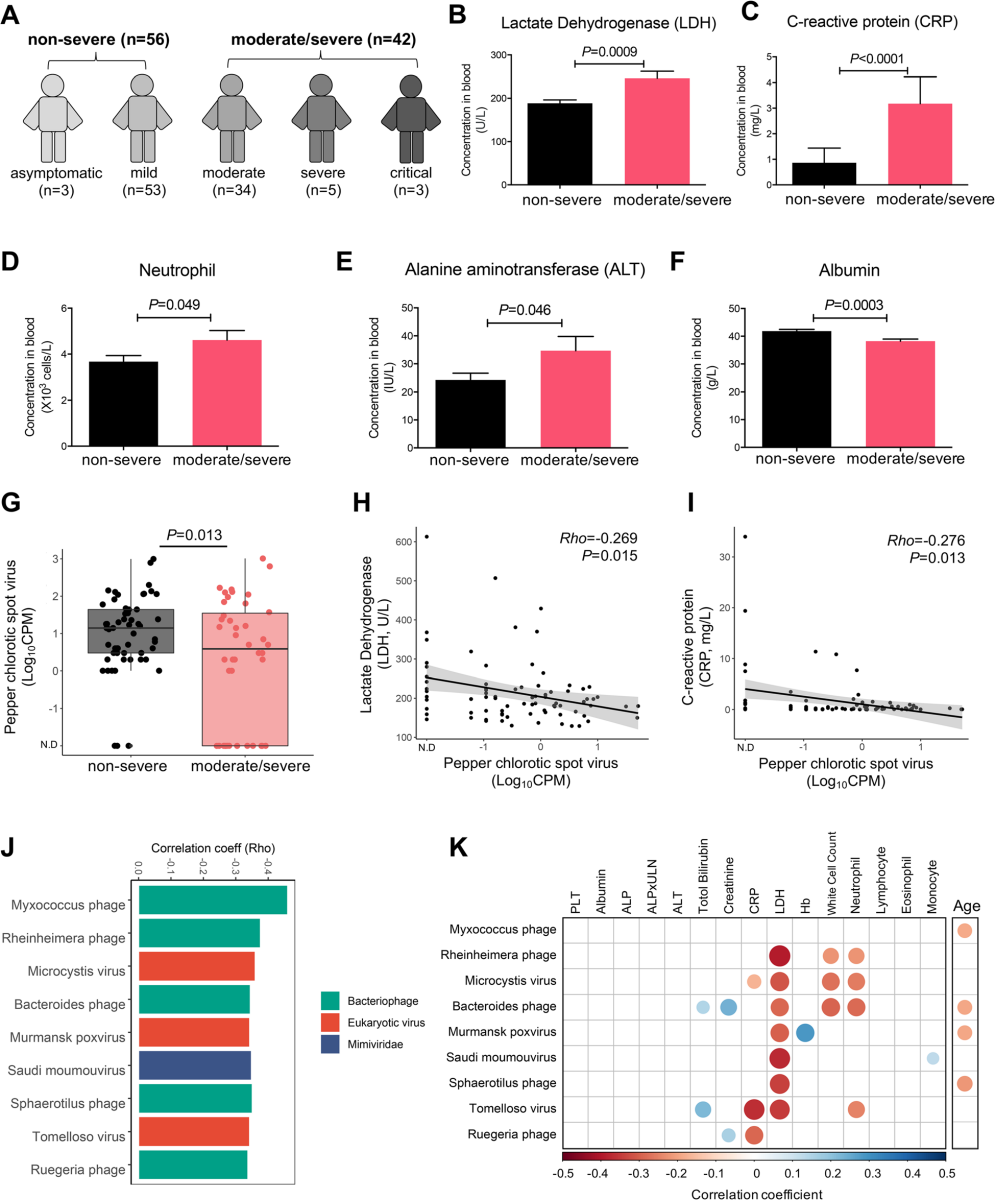
Fecal virus species correlated with COVID-19 severity and blood parameters. **a** Recruited COVID-19 patients and their symptom severity. Patients were separated in to non-severe (*n*=56) and moderate/severe (*n*=42) groups. **b**–**f** Blood measurement results for LDH, C-reactive protein, neutrophil count, ALT, and albumin concentrations. Data are shown in mean ± s.e. The comparisons were made between non-severe and moderate/severe groups via Mann-Whitney test. **g**–**i** The abundance of pepper chlorotic spot virus in fecal RNA virome and its association with disease severity (**g**), blood concentrations of LDH (**h**), and C-reactive protein (**i**). CPM, count per million reads. Between-two group comparison was conducted by Mann-Whitney test. Correlation test was performed by Spearman correlation test. **j**, **k** The 9 DNA virus species that negatively correlated with severity of COVID-19 (**j**) and their correlations with blood measurements (**k**). Correlation test was performed by Spearman correlation test. **k** The color and intensity denote the spearman correlation direction and coefficient, where only the significant correlations with blood parameters of |correlation coefficient| > 0.3 were shown

**Fig. 6:**
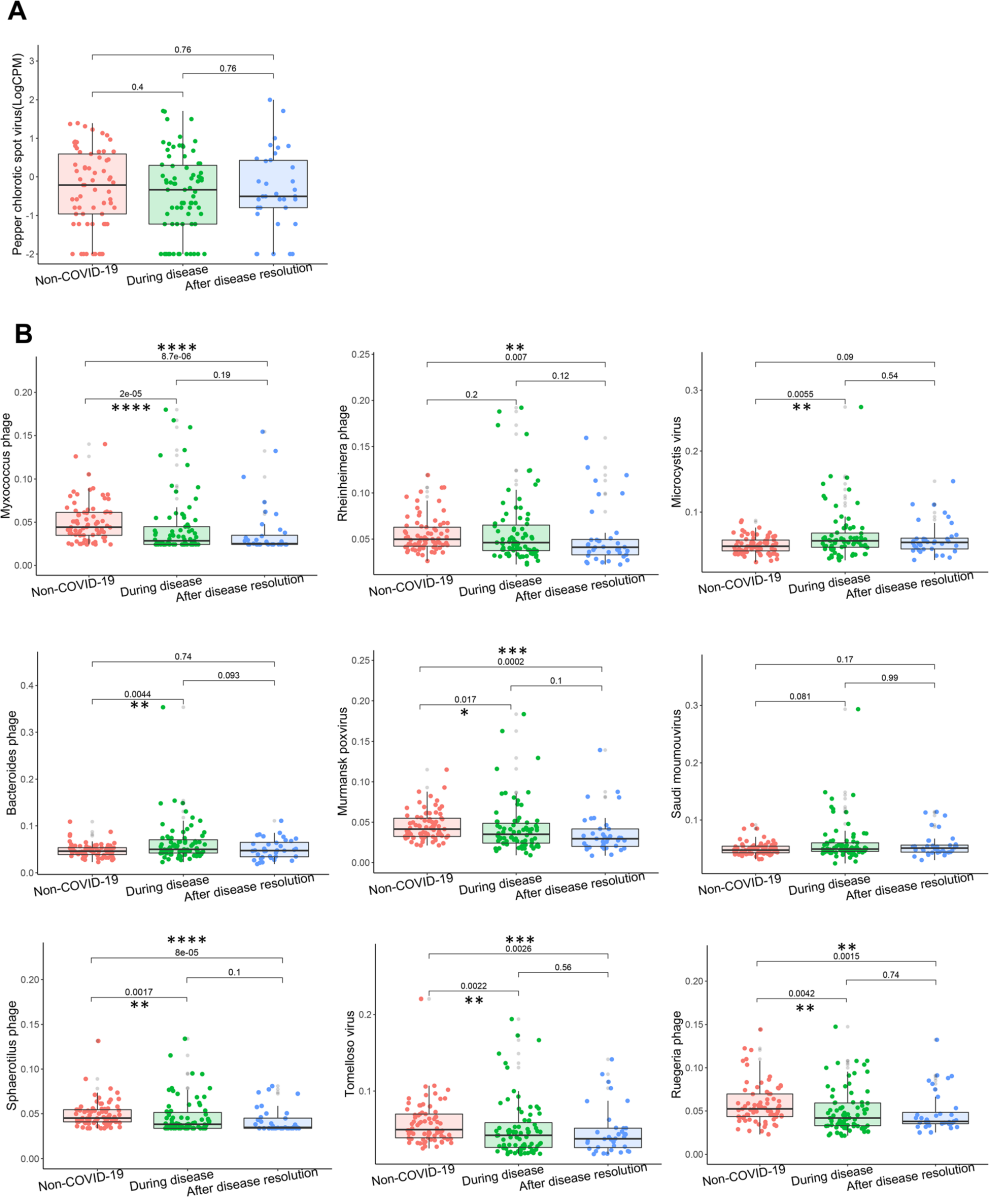
Longitudinal changes in the abundance of RNA (**a**) and DNA viruses (**b**) that correlated with COVID-19 severity (shown in Fig. 3) in feces of COVID-19 patients during disease course and after disease resolution, as compared to non-COVID-19 patients. The abundance of RNA virus was expressed in Log_10_CPM, where CPM denotes count per million reads. The abundance of DNA virus was expressed in normalized abundance (DESeq adjusted RPKM), where RPKM denotes reads per kilobase per million mapped reads. Statistical significance was calculated by Man-Whitney test with *p<0.05, **p<0.01, ***p<0.001, and ****p<0.0001

Based on COVID-19 disease symptoms and severity classification criteria [20], we stratified our patients into non-severe (*N*=56; asymptomatic/mild cases) and moderate/severe groups (*N*=42; moderate/severe/critical cases) (Fig. 5a). Compared to non-severe cases, moderate/severe cases showed a significantly higher blood levels of LDH, neutrophil count, C-reactive protein (CRP), alanine aminotransferase (ALT), and lower blood levels of albumin at admission (all *p*<0.05, Fig. 5b–f, Supplementary Figure 5). Our data are in line with recent reports highlighting that more severe cases had more pronounced systemic inflammatory responses [2, 21–24]. We then explored association between baseline fecal RNA and DNA virome profiles with COVID-19 severity and blood measurements at hospitalization. Abundance of the plant-derived RNA virus, pepper chlorotic spot virus (PCSV) was higher in patients with non-severe than those with moderate/severe disease (*p*=0.013, Fig. 5g). In addition, a high abundance of PCSV in feces was associated with low blood concentrations of the inflammation markers, LDH and CRP (correlation coefficient Rho=−0.269 and −0.276, respectively, Fig. 5h, i). Similarly, abundance of 9 DNA virus species (Myxococcus phage, Rheinheimera phage, Microcystis virus, Bacteroides phage, Murmansk poxvirus, Saudi moumouvirus, Sphaerotilus phage, Tomelloso virus, and Ruegeria phage) in feces negatively correlated with COVID-19 severity (all FDR *p*<0.05, Fig. 5j). In particular, 8 out of the 9 DNA virus species showed strong negative correlation with blood levels of the inflammation indicators LDH, neutrophil count, white cell count, or CRP (Fig. 5k). Interestingly, among them, four viral species, Myxococcus phage, Bacteroides phage, Murmansk poxvirus, and Sphaerotilus phage, which inversely associated with inflammation indicators, also inversely correlated with host age (Fig. 5k). This result coincides with the observation that elderly individuals were at higher risk for unfavorable severe COVID-19 outcomes [2, 25]. These data suggest that such RNA and DNA viruses may counteract the effect of SARS-CoV-2 infection predisposing infected subjects to a less severe COVID-19 course. Five out of the 9 severity-associated DNA virus species showed persistent lower abundances in the feces of COVID-19 patients during disease course and after disease resolution compared to non-COVID-19 controls (all *p*<0.05, Fig. 6), indicating an unfavorable effect of SARS-CoV-2 infection on these gut viruses. The cause or consequence of such associations needs to be further explored. In addition, a large number of DNA virus species in feces (*n*=132) showed significant correlations with blood parameters in COVID-19 patients, most of which were negative correlations with blood LDH concentrations, neutrophil, and white cell counts (Supplementary Figure 6). These data underscore the potential significance of gut DNA virome in calibrating host immunity and counteracting infection of SARS-CoV-2, warranting further investigation.

## Discussion

In this study, we provide the first comprehensive characterization of both the gut RNA and DNA virome in patients with COVID-19 and explore the association between host gut virome features with COVID-19 severity. Diet-originated plant RNA viruses are known to be prevalent in a healthy human gut [26]. In the present study, two pepper-derived RNA virus species were found to be underrepresented (PMMoV) or correlated inversely with disease severity in COVID-19 patients (PCSV). In addition, COVID-19 patients had higher abundances of eukaryotic- and environment-derived viruses whereas non-COVID-19 subjects harbored more bacteriophages in feces. Such virome compositional features might be a consequence of SARS-CoV-2 infection along with its impact on the host immunity and the holistic ecology of the host gut microbiota [8, 15, 16]. The gut virome changes in COVID-19 were modest, given that COVID-19 is an acute respiratory disease primarily inflicting the respiratory tract with miniscule enteric involvement [2–5] and that human gut virome is substantially diverse across healthy individuals [27]. We also observed persistently discrepant viromes in COVID-19 patients with that of healthy controls over time, both during disease course and after disease resolution. In a subset of COVID-19 patients, even more dissimilar virome configurations to that of healthy controls at the last follow-up were observed. Concordantly, a study has shown that a proportion (~35%) of COVID-19 patients reported not returning to their usual state of health after hospital discharge, manifesting fatigue, and dyspnea [28]. Such data indicates that SARS-CoV-2 infection may have long-term detrimental effect on certain COVID-19 patients. Together, the persistently different gut RNA and DNA viromes in COVID-19 versus healthy controls, during disease course and after disease resolution, imply a potential long-lasting detrimental effect of SARS-CoV-2 infection on the host.

The immediate response to any infectious-disease outbreak is to approach it from the pathogen perspective because disease severity is assumed to be a direct function of pathogen burden [29]. However, the complexities of SARS-CoV-2 infection serve as an important reminder that this perspective is not sufficient for understanding the survival of infectious diseases [30, 31]. Disease and immune tolerance as opposed to hyper-inflammatory (cytokine storm) state is proposed to be critical for limiting damage to the host and for improving patient survival in COVID-19 [31–33]. In line with findings from recent studies probing blood immune cell and biochemical measurements in COVID-19 patients [21–23], we found elevations of innate immune cells (neutrophils and white cells) and pro-inflammatory indicators (C-reactive protein and LDH) in the blood of more severe COVID-19 cases. Of note, fecal SARS-CoV-2 levels were significantly higher in asymptomatic/mild COVID-19 patients than in patients with a moderate disease course. This might be due to an immune-tolerant phenotype in mild COVID-19 patients which accommodates non-symptomatic existence of viruses in the host, rather than fulminant host immune defense against SARS-CoV-2 which results in viral attenuation but host tissue damage in severer COVID-19 patients. We did not find significant linear correlation between fecal SARS-CoV-2 levels and COVID-19 severity, yet a large number of gut prokaryotic and eukaryotic viruses correlated negatively with blood concentrations of immune cells and pro-inflammatory proteins and COVID-19 severity, suggesting that gut virome may calibrate host physiology and immune response against SARS-CoV-2 infection.

The bacteriophages, Escherichia virus (phage) and Enterobacter phage, were also enriched in COVID-19 patients. Expansion of these phages has been causally implicated in gut inflammation and host interferon response in mice and humans [14, 19]. These data highlight that the enteric virome alteration in COVID-19 may further contribute to immunological and physiological changes in the host during the disease course. In our study, we observed administration of Lopinavir-ritonavir was associated with decreased abundance of Listeria phage, a phage infecting the pathogenic Listeria (bacteria). It suggests that use of antivirals may tune host bacteriophage-bacteria ecology in the gut, beyond its role in enhancing host immune defense against infectious viruses.

Interestingly, among the identified 9 viral species inversely correlated with disease severity of COVID-19 and inflammation markers in the blood, 4 (Myxococcus phage, Bacteroides phage, Murmansk poxvirus, and Sphaerotilus phage) also inversely correlated with human age. This finding may partly explain the observation that elderly individuals are prone to unfavorable severe COVID-19 outcomes [2, 25] and shed light on the importance of gut viruses in human pathophysiology. Those phages associated with a mild COVID-19 course and younger human age might be leveraged for therapeutic exploration.

The human gut RNA virome, comprising multiple plant-related viruses, are both compositionally and functionally under-explored. The question of whether plant viruses can infect humans remains unresolved, though anecdotal evidence suggests so [34]. PMMoV is the most abundant and prevalent RNA virus infecting pepper in human feces, which is proposed to be a viral indicator for human fecal pollution in aquatic environments and water treatment systems [17]. Current literature favors that it unlikely to infect human cells. Its relationships with human immune/physiological responses are also unknown. Analogous to the changes in the bacteriophage fraction of the gut virome in COVID-19, alterations in the plant-related RNA virome fraction in COVID-19 might also be a consequence of SARS-CoV-2 infection. SARS-CoV-2 may modulate host immunity to create an unfavorable environment for certain RNA viruses, such as PMMoV. In return, the demise or lysis of such viruses in human gut could result in a release of nucleic acids, proteins, and lipids that serve as pathogen-associated molecular patterns (PAMP) that trigger inflammatory responses to induce cellular infiltration, cytokine production, and even tissue damage. Though study has shown that some plant viruses are much resistant and can remain long time under the harsh GI conditions [35], data are lacking about the transit time of various plant-related viruses through the human GI tract, particularly for those viruses correlated with COVID-19.

A population-based study from France reported that PMMoV was detected with a low prevalence in hospitalized adults (7%) and children (<1%) [36]. In contrast, PMMoV is much more prevalent in our cohort (~67%) and another US cohort [26]. Human gut virome is known to vary substantially across population, geography and lifestyle [27], and highly individual-specific [13]. Therefore, the observed differences in gut virome configuration between studies might be accounted for by the host and environmental factors in conjunction with population characteristics.

Over the long co-evolutionary history of viruses with their hosts, the parasitic viruses are able to snatch genetic elements/remnants form their hosts during infection and/or co-existence [37, 38]. This is particularly true for bacteriophages and their bacteria hosts. Phages are genetically diverse and their genome architectures are characteristically mosaic, driven by horizontal gene transfer (HGT) with other phages and host genomes [39, 40]. Therefore, the genomes of phages are highly complex and diverse, and they are composed of genes with distinct and varied evolutionary histories [41, 42]. For instance, numerous cultivated and uncultivated viruses encode ribosomal proteins [43]. In addition, bacteria-encoded tRNA synthase was also found in phages [44]. Concordantly, certain ribosomal protein- and tRNA synthase-coding genes were enriched in COVID-19 viromes seen in our study. Overall, the enrichment of bacterial genes in COVID-19 virome may partly be ascribed to the co-evolution nature of virus-host relationship or the bacterial dysfunction in COVID-19.

There are some limitations of this study. First, it is exploratory in nature without clear cause or consequence effect established. Confirmation of gut virome alterations and their impact on disease severity or trajectory will require functional validation in other populations and animal studies. Second, stool collected after hospitalization for virome analysis does not represent the bona fide baseline microbiome before or at COVID-19 onset. Further studies should prospectively include healthy subjects, and if infected with SARS-CoV-2, followed-up at disease onset, during disease course, and long term after discovery, to delineate the role of virome changes in SARS-CoV-2 infection, severity, and post-infection recovery. Third, we documented the dietary record of patients over the course of hospitalization, yet did not capture the diet record before disease onset nor that of non-COVD-19 controls, which precludes us to investigate the virtual effect of diet on the gut virome differences between COVID-19 and non-COVID-19 cases. However, the diets provided during time of patient hospitalization were diverse as opposed to monotonous and were consistent with habitual daily diet consumed by Hong Kong Chinese. In addition, diets over the course of hospitalization did not show significant effects in the temporal variation of the gut DNA or RNA virome of hospitalized COVID-19 patients. Moreover, most of the viruses differed between COVID-19 patients and non-COVID-19 subjects were persistently different over the course of hospitalization, despite various diets were provided during time of hospitalization. Altogether, it is likely that the virome alterations in COVID-19 patients are at least partially reflective of the disease course rather than an effect of diet.

## Conclusions

In summary, both enteric RNA and DNA virome were different in COVID-19 versus non-COVID-19 subjects, which persisted even after disease resolution. Gut virome may calibrate host immunity and regulate severity to SARS-CoV-2 infection. Our observation that gut viruses inversely correlated with both severity of COVID-19 and host age may partly explain that older subjects are prone to severe and unfavorable COVID-19 outcomes. Our data altogether highlight the significance of human gut virome in COVID-19 disease course and potentially therapeutics.

## Materials and methods

### Study subject and design

This prospective study enrolled 98 hospitalized patients with COVID-19 and 78 gender- and co-morbidity-matched non-COVID-19 controls (Table 1 and Supplementary Table 1). All study subjects had a single stool specimen collected at inclusion. All COVID-19 patients had blood samples collected at admission and measured for white cells and biochemical markers in plasma (Supplementary Table 1). Among the included COVID-19 patients, 37 COVID-17 patients had longitudinal follow-up from hospitalization till after discharge and had serial stool specimens collected (2–3 times per week, Supplementary Figure. 1). SARS-CoV-2 infection was confirmed by two consecutive RT-PCR test targeting different regions of the RdRp gene performed by the local hospital and Public Health Laboratory Service. All COVID-19 patients were admitted to the Prince of Wales Hospital or the United Christian Hospital, Hong Kong, from February through May, 2020. COVID-19 severity was categorized as (i) asymptomatic, if there was no clinical symptoms; (ii) mild, if there was no radiographic evidence of pneumonia and the clinical symptoms were light; (iii) moderate, if fever, respiratory tract and other symptoms present and imaging suggests pneumonia; (v) severe, if respiratory rate ≥30/min, or oxygen saturation ≤93% when breathing ambient air; or PaO_2_/FiO_2_ ≤ 300 mmHg (1mmHg = 0.133 kPa); or (v) critical, if there was respiratory failure requiring mechanical ventilation, shock, or organ failure requiring intensive care [20]. All hospitalized patients were provided 3 standardized meals daily, varying from Monday to Sunday, by the department of hospital catering service (Supplementary Table 2). The dietary component and pattern were consistent with the habitual diets commonly consumed by Hong Kong Chinese.

Non-COVID-19 controls were average healthy Hong Kong individuals recruited at Prince of Wales Hospital before the COVID-19 outbreak and tested negative for SARS-CoV-2 during the COVID-19 pandemic. The inclusion criteria for non-COVID-19 subjects are (1) aged ≥18 years old, (2) competent to provide informed consent (no mental illness or dementia), (3) have no underlying infectious or acute disease, and (4) have lived in the same geographic area for the preceding 6 months. The exclusion criteria for non-COVID-19 subjects are (1) the use of laxatives or anti-diarrheal drugs in the last 3 months; (2) recent dietary changes (e.g., becoming vegetarian/vegan); (3) known complex infections or sepsis (excluding uncomplicated infections such as influenza); (4) known history of severe organ failure (including decompensated cirrhosis, malignant disease, kidney failure, epilepsy, active serious infection, acquired immunodeficiency syndrome); (5) bowel surgery in the last 6 months (excluding colonoscopy/procedure related to perianal disease); (6) presence of an ileostomy/stoma; (7) current pregnancy; and (8) colonoscopy in the last month prior to sampling. This study was approved by the Joint Chinese University of Hong Kong–New Territories East Cluster Clinical Research Ethics Committee (Reference number: 2020.076). All subjects provided informed consent to participate in this study and agreed for publication of the research results. Data including demographic, epidemiological, clinical, laboratory results, treatment, and medication were extracted from the electronic medical records in Hong Kong Hospital Authority clinical management system. This study was conducted in accordance with the Declaration of Helsinki.

### Fecal viral DNA and RNA extraction, isolation, and shotgun metagenomics

The total viral nucleic acid was extracted from fecal sample, using TaKaRa MiniBEST Viral RNA/DNA Extraction Kit (Takara, Japan) following manufacturer’s instructions. Extracted total viral nucleic acid was then purified by the DNA and RNA Clean & Concentrator Kits (Zymo Research, CA, USA) to obtain viral DNA and RNA, respectively. After the quality control procedures by Qubit 2.0, agarose gel electrophoresis, and Agilent 2100, the qualified DNA and RNA were subjected to library preparation using Nextera DNA Flex Library Preparation kit (Illumina, USA) and KAPA RNA HyperPrep Kit (Roche, Swiss). The qualified libraries were then sequenced (150bp paired end) on Illumina Novaseq platform.

### RNA virome classification and quantification

Raw RNA virome metagenomic sequence reads were filtered and quality-trimmed using Trimmomatic v0.36 [45] as follows: (1) trimming low-quality base (quality score < 20), (2) removing reads shorter than 50bp, and (3) removing sequencing adapters. Contaminating human reads were filtering using Kneaddata (Reference database: GRCh38 p12) with default parameters. Cleaned RNA viral reads were rarefied to even depth (20 million reads per sample) and profiled via Kraken2 [46] against the NCBI viral Refseq database as of April 20, 2020. Kraken2 has demonstrated appropriate performance in viral metagenomic classification for time-constrained viral diagnostics and profiling purposes, as well as for surveillance and outbreak source tracing [47]. The abundance of constituent RNA virus species was calculated as count per million sequencing reads (CPM). A CPM filter of > 1 was used to select species for downstream differential analysis. Differential viral taxa between COVID-19 patients and non-COVID-19 controls were identified using Multivariate Association with Linear Models (MaAsLin2), while adjusting for confounding factors.

### DNA virome classification and quantification

Raw sequence reads were filtered utilizing Trimmomatic using the following parameters; SLIDINGWINDOW: 4:20, MINLEN: 60 HEADCROP 15; CROP 225. Contaminating human reads were filtering using Kneaddata (Reference database: GRCh38 p12) with default parameters. Megahit, with default parameters, was chosen to assemble the reads into contigs per sample. Assemblies were subsequently pooled and retained if longer than 1kb. Bacterial contigs were removed by using an extensive set of inclusion criteria to select viral sequences only. Briefly, contigs were required to fulfill one of the following criteria: (1) categories 1–6 from VirSorter when running with default parameters and Refseqdb (–db 1) positive, (2) circular, (3) greater than 3kb with no BLASTn alignments to the NT database (*e* value threshold: 1e−10), (4) a minimum of 2 pVogs with at least 3 per 1kb, (5) BLASTn alignments to viral RefSeq database (v.89) (*e* value threshold: 1e−10), and (6) less than 3 ribosomal proteins as predicted using the COG database. HMMscan was used to search the viral contig ORFs against the pVOGs hmm profile database with an *e* value filter of 1e−5, retaining the top hit in each case. Afterwards, a fasta file combining viral contigs was compiled. This viral database includes the viral contigs recovered by the screening criteria from the bulk metagenomic assemblies. Then, the paired reads were mapped to the viral contig database with BWA, using default parameters. The viral operational taxonomic unit (OTU) table of viral abundance was pulled from BWA sam output files by customized script and normalized by the number of metagenomic reads (metagenomic reads were rarefied to even depth, 20 million reads per sample). The contigs were then subjected to open reading frame (ORF) prediction, using MetaProdigal (v2.6.3) with the metagenomics procedure (-p meta). To annotate the predicted ORFs, the amino acid sequences of the ORFs were queried by Diamond against the viral RefSeq protein (v84) with an *e* value <10^˗5^ and a bitscore >50. The viral Refseq proteins with the top closest homologies (*e* value <10^˗5^ and bitscore >50) were considered for each ORF. Contigs were taxonomically binned to a taxon according to the predominant assignment of its constituent ORFs. Contigs shared the same taxonomic identity were therefore collapsed. The viral contig abundances in each sample were finally summed as reads per kilobase per million mapped reads (RPKM, shown in Supplementary Table 3). The virome community differences were visualized via PCoA analysis based on the Euclidean distance between each individual’s virome. Differential DNA viral taxa between COVID-19 patients and non-COVID-19 controls were identified using DESeq, while adjusting for confounding factors.

### Virome functionality analysis

Viral reads were retrieved via BBMap against the collection of assembled viral contigs, followed by functionality enquiry and annotation via *HUMANN2 v0.9.4*. Predicted functions were collapsed by gene family identity, with abundance values expressed in RPK (reads per kilobase). To establish the presence or absence a function within a sample, a stringent RPK threshold value > 10 was used to be defined as present. Differential functions between COVID-19 and non-COVID-19 subjects were identified by DESeq, while adjusting for co-morbidities, antiviral, and age.

### Detection of fecal SARS-CoV-2 viral load

SARS-CoV-2 viral loads in stool and nasopharyngeal swab specimens were measured using real-time reverse-transcriptase-polymerase chain-reaction (RT-PCR) assay. Viral RNA from specimens was extracted using QIAamp Viral RNA Mini Kit (Qiagen, Hilden, Germany). SARS-CoV-2 RNA was quantified using real-time reverse-transcriptase-polymerase-chain-reaction (RT-PCR). The primer-probe set N1 (2019-nCoV_N1-F: 5’-GAC CCC AAA ATC AGC GAA AT-3’, 2019-nCoV_N1-R: 5’-TCT GGT TAC TGC CAG TTG AAT CTG-3’ and 2019-nCoV_N1-P: 5’-FAM-ACC CCG CAT TAC GTT TGG TGG ACC-BHQ1-3’) designed by the US Centers for Disease Control and Prevention (CDC) was purchased from the Integrated DNA Technologies, USA. The one-step real-time RT-PCR reaction contained 10 μL of the extracted preparation, 4 μL T*aq*Man™ Fast Virus 1-Step Master Mix (Applied Biosystems, USA) in a final reaction volume of 20 μL. The primer and probe concentration were 0.5 μM and 0.125 μM, respectively. The cycling conditions, 25°C for 2 min, 50°C for 15 min, 95 °C for 2 min, followed by 45 cycles of 95 °C for 15 s, and 55 °C for 30 s, were performed with the StepOnePlus Real-Time PCR System (Applied Biosystems, USA). The cycle threshold (Ct) values of real-time RT-PCR were converted into viral RNA copies based on a standard curve prepared from 10-fold serial dilutions of know copies of plasmid containing the full N gene (2019-nCoV_N_Positive Control, Integrated DNA Technologies, USA). Samples were considered as negative if the Ct values exceeded 39.9 cycles.

### Correlation between viruses, COVID-19 severity, and blood measurements

Spearman correlation analyses were conducted to correlate both RNA and DNA virome abundance profiles with blood parameter measurements. Spearman correlation analyses with multiple comparison adjustment were conducted to correlate the DNA virome abundance profile with COVID-19 severity across COVID-19 patients.

## Supplementary Information

**Additional file 1: Supplementary Figure 1.** Schematic diagram of COVID-19 patient (n=37) follow-up, including disease onset, admission, stool sample collection, duration of hospital stay. “CoV” denotes patient with COVID-19. Stool specimens were serially collected for separate shotgun metagenomic sequencing of RNA and DNA virome; “SARS-CoV-2 PCR negative in nasopharyngeal test”: the first negative result for SARS-CoV-2 virus in two consecutive negative nasopharyngeal tests, upon which patient was then discharged.

**Additional file 2: Supplementary Figure 2.** Temporal changes of the RNA viruses, SARS-CoV-2 (A) and PMMoV (B), in the faecal RNA virome in each COVID-19 case. CPM, count per million reads.

**Additional file 3: Supplementary Figure 3.** SARS-CoV-2 viral load. Stool SARS-CoV-2 viral load in COVID-19 patients were detected by quantitative RT-PCR (A) and RNA virome shotgun metagenomics sequencing (B) respectively. Between-group comparison was conducted by one-way *anova*, **p<0.01, *p<0.05. (C) Comparison of SARS-CoV-2 viral loads between nasopharyngeal swab and fecal specimens. Statistical analysis was conducted by Mann-Whitney test. (D) Correlation of levels of SARS-CoV-2 viral load between nasopharyngeal swab and fecal specimens, calculated by *Pearson* correlation analysis.

**Additional file 4: Supplementary Figure 4.** Heatmap of correlations of COVID-19-enriched faecal viral functions and species. The color and intensity denote the spearman correlation direction and coefficient, where only the significant correlations were shown. Viruses labeled with asterisk were species enriched in COVID-19. Only the most abundant 80 species in faecal virome were plotted and ranked in descending order (from left to right) on the basis of the relative abundance.

**Additional file 5: Supplementary Figure 5.** Blood parameter levels in COVID-19 patients between non-severe and moderate/severe groups. Data are shown in mean±s.e. Statistical significance was performed by Mann-Whitney test.

**Additional file 6: Supplementary Figure 6.** The faecal DNA viruses at patient baseline correlated with blood parameters in COVID-19 patients. The color and intensity denote the spearman correlation direction and coefficient, where only the significant correlations with |correlation coefficient| > 0.3 were shown.

**Additional file 7: Supplementary Table 1.** Subject characteristics and blood measurements.

**Additional file 8: Supplementary Table 2.** Diet regimen during time of hospitalization.

**Additional file 9: Supplementary Table 3.** Viral abundance profile in faecal samples. Abundance is expressed in RPKM.

## Data Availability

Virome metagenomic sequencing data has been deposited to the NCBI Sequence Read Archive under BioProject accession number PRJNA657711.
