# Supplementary figures and images for: Temporal landscape of human gut RNA and DNA virome in SARS-CoV-2 infection and severity
Source: Microbiome. 2021 Apr 14;9:91. doi: 10.1186/s40168-021-01008-x (PMC8044506; doi:10.1186/s40168-021-01008-x)

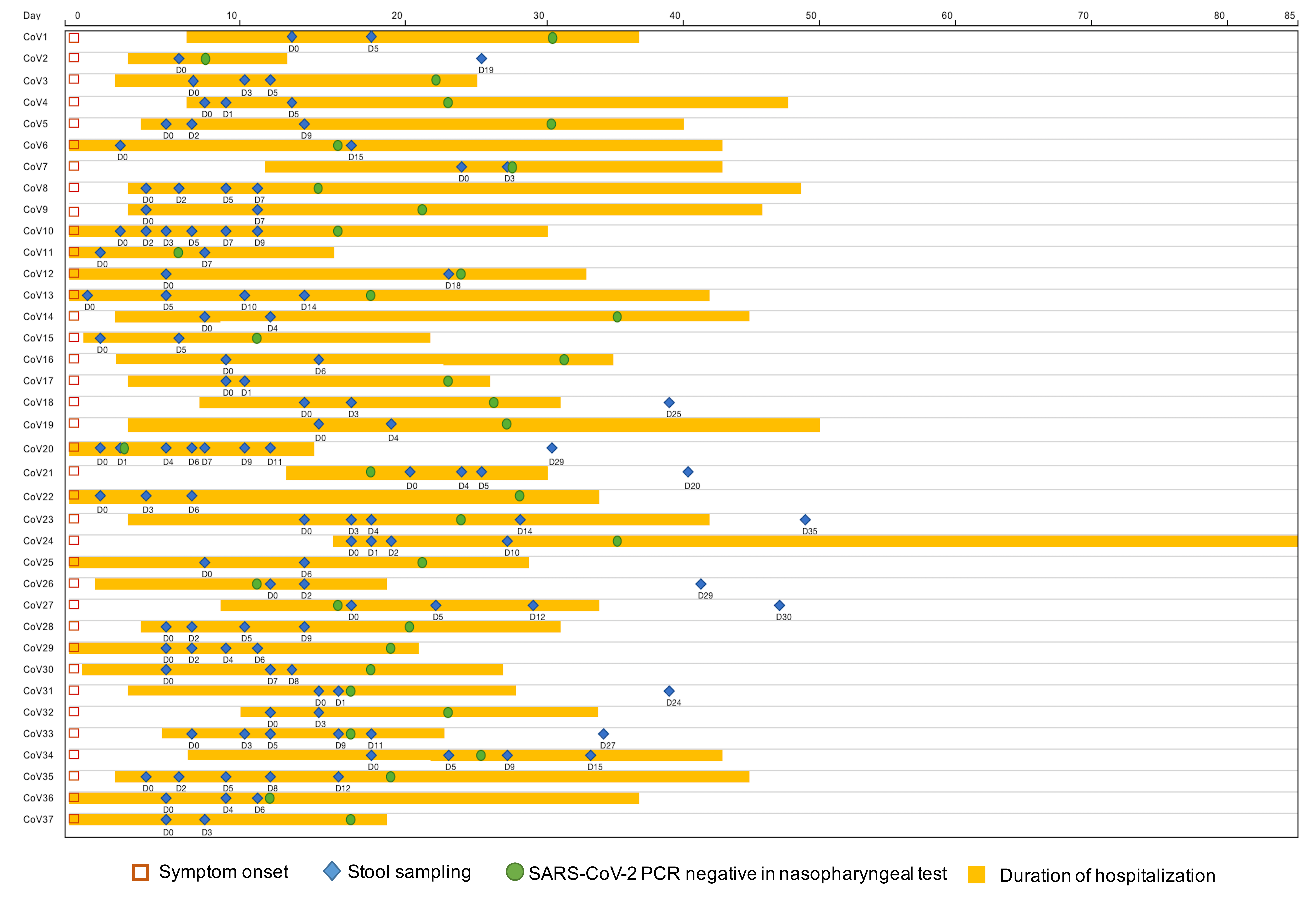

Supplement: Supplementary file 2 — Additional file 1: Supplementary Figure 1. Schematic diagram of COVID-19 patient (n=37) follow-up, including disease onset, admission, stool sample collection, duration of hospital stay. “CoV” denotes patient with COVID-19. Stool specimens were serially collected for separate shotgun metagenomic sequencing of RNA and DNA virome; “SARS-CoV-2 PCR negative in nasopharyngeal test”: the first negative result for SARS-CoV-2 virus in two consecutive negative nasopharyngeal tests, upon which patient was then discharged. [file 40168_2021_1008_MOESM2_ESM.tif]

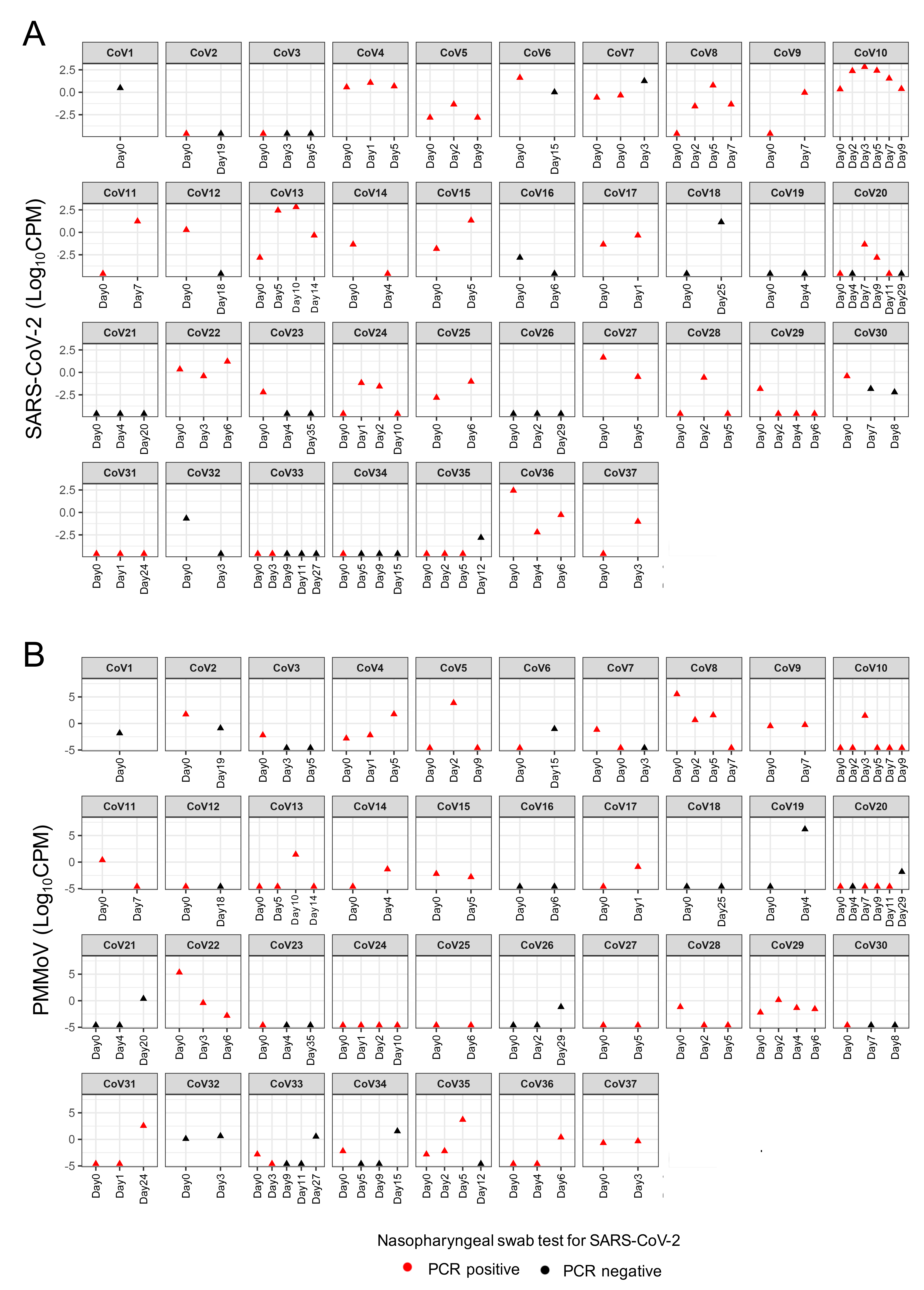

Supplement: Supplementary file 3 — Additional file 2: Supplementary Figure 2. Temporal changes of the RNA viruses, SARS-CoV-2 (A) and PMMoV (B), in the faecal RNA virome in each COVID-19 case. CPM, count per million reads. [file 40168_2021_1008_MOESM3_ESM.tif]

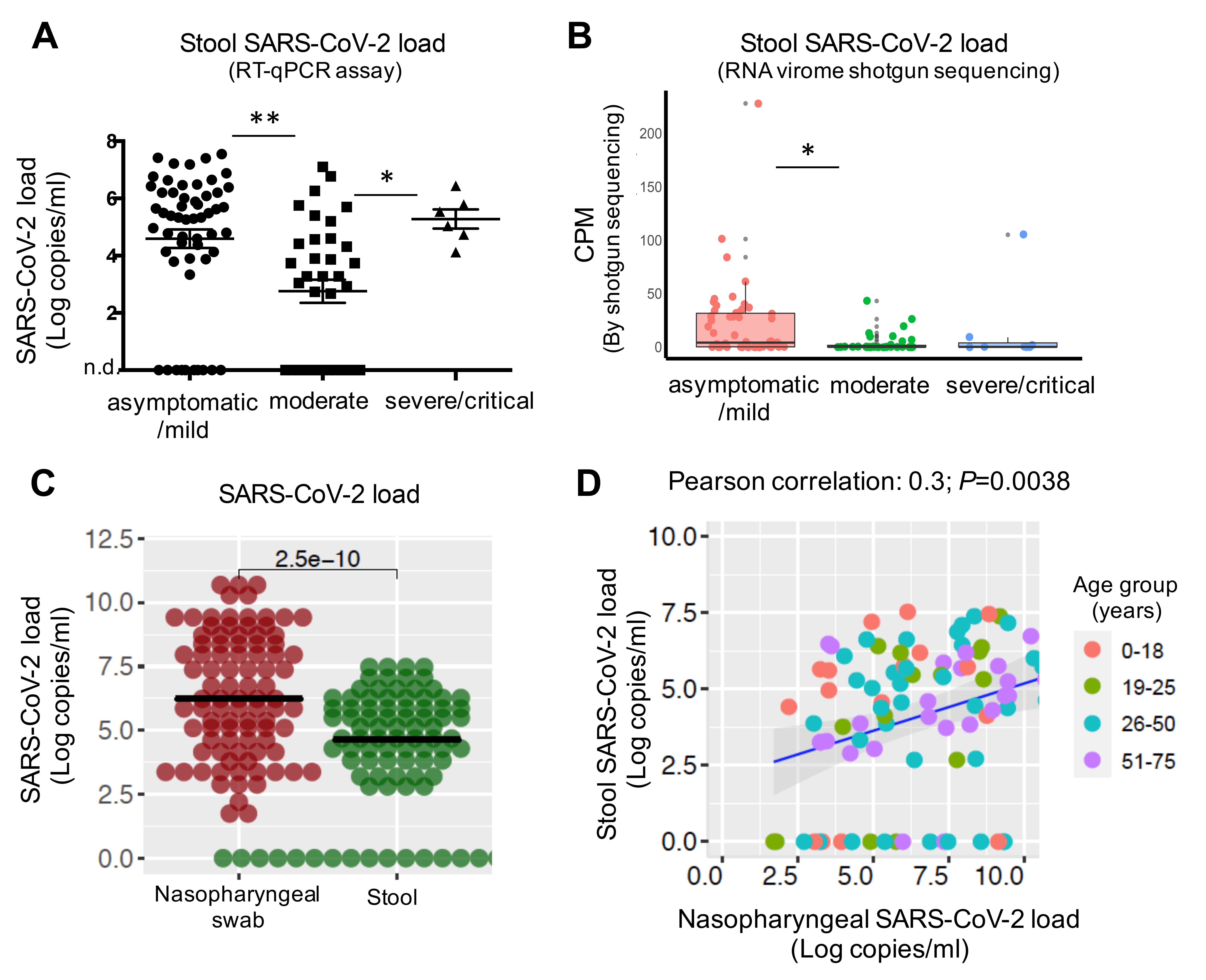

Supplement: Supplementary file 4 — Additional file 3: Supplementary Figure 3. SARS-CoV-2 viral load. Stool SARS-CoV-2 viral load in COVID-19 patients were detected by quantitative RT-PCR (A) and RNA virome shotgun metagenomics sequencing (B) respectively. Between-group comparison was conducted by one-way anova, **p<0.01, *p<0.05. (C) Comparison of SARS-CoV-2 viral loads between nasopharyngeal swab and fecal specimens. Statistical analysis was conducted by Mann-Whitney test. (D) Correlation of levels of SARS-CoV-2 viral load between nasopharyngeal swab and fecal specimens, calculated by Pearson correlation analysis. [file 40168_2021_1008_MOESM4_ESM.tif]

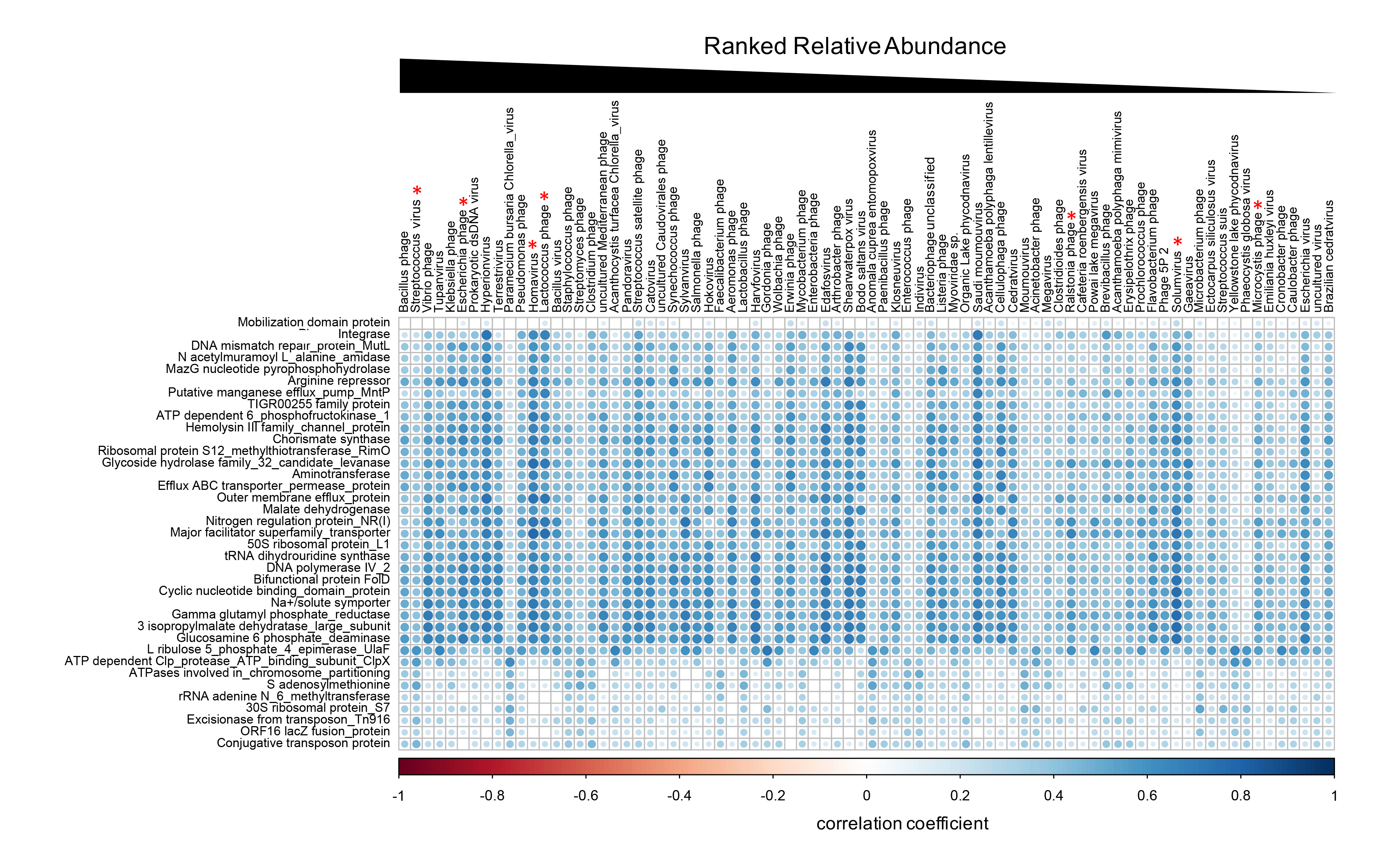

Supplement: Supplementary file 5 — Additional file 4: Supplementary Figure 4. Heatmap of correlations of COVID-19-enriched faecal viral functions and species. The color and intensity denote the spearman correlation direction and coefficient, where only the significant correlations were shown. Viruses labeled with asterisk were species enriched in COVID-19. Only the most abundant 80 species in faecal virome were plotted and ranked in descending order (from left to right) on the basis of the relative abundance. [file 40168_2021_1008_MOESM5_ESM.tif]

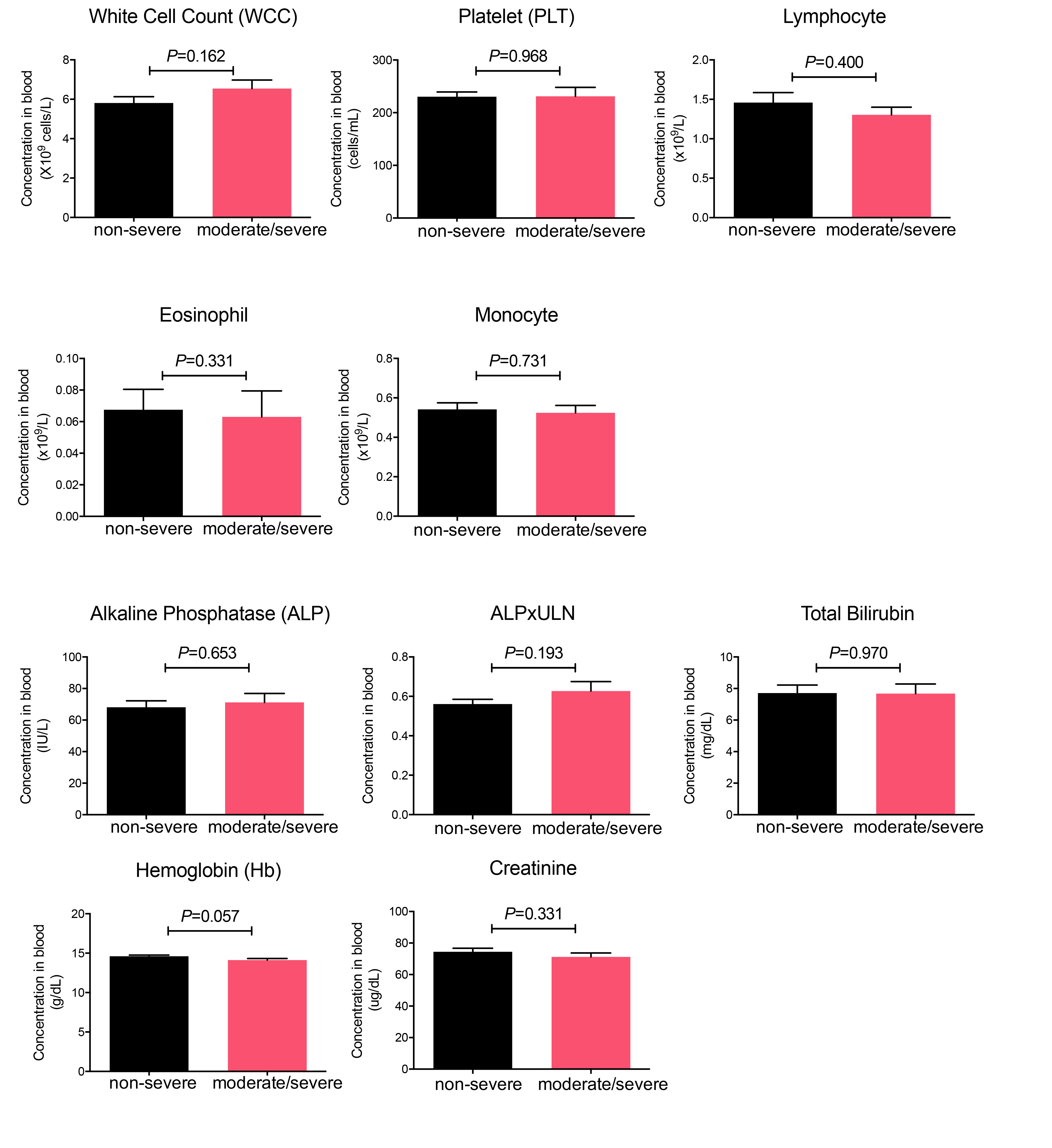

Supplement: Supplementary file 6 — Additional file 5: Supplementary Figure 5. Blood parameter levels in COVID-19 patients between non-severe and moderate/severe groups. Data are shown in mean±s.e. Statistical significance was performed by Mann-Whitney test. [file 40168_2021_1008_MOESM6_ESM.tif]

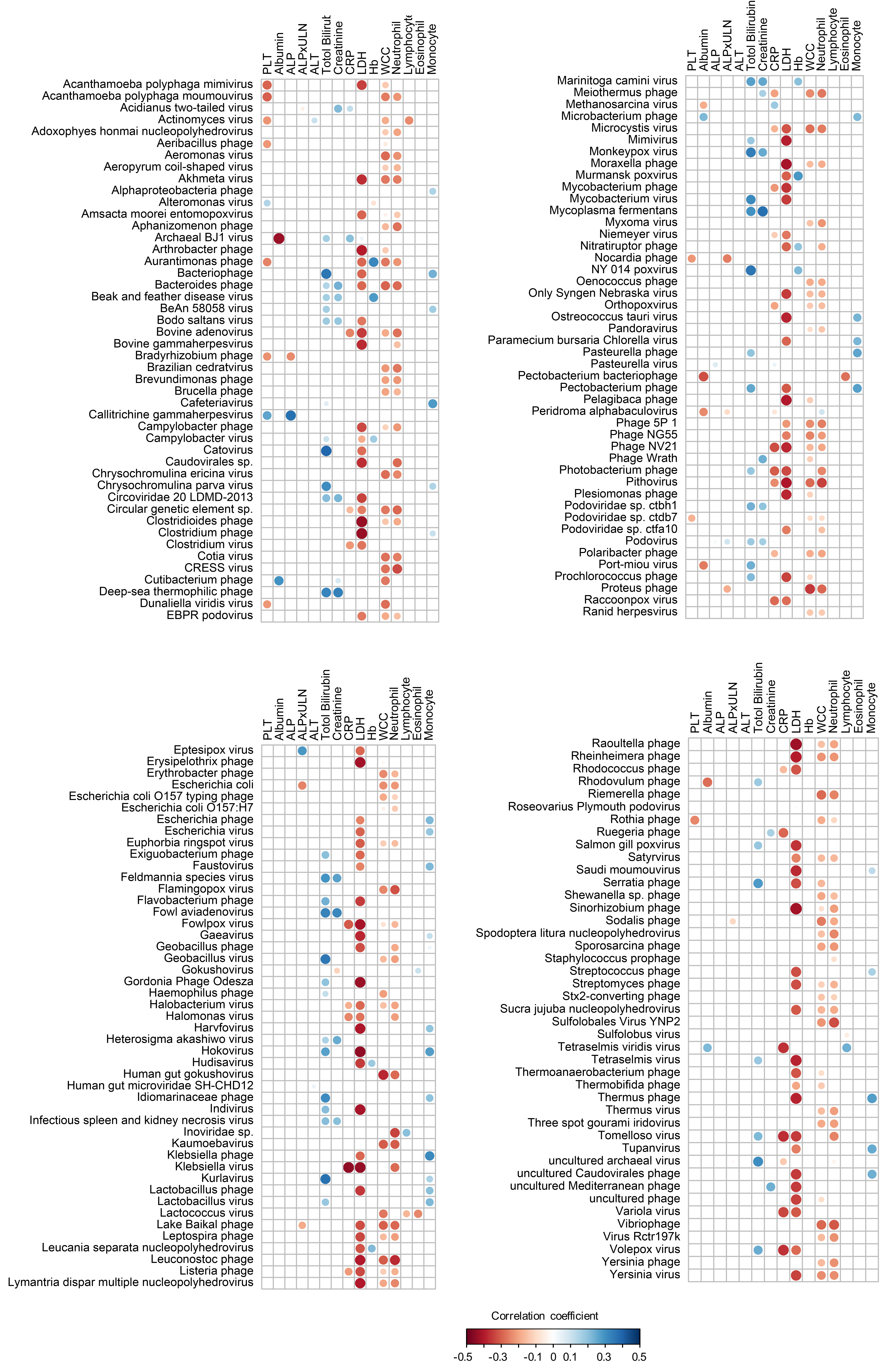

Supplement: Supplementary file 7 — Additional file 6: Supplementary Figure 6. The faecal DNA viruses at patient baseline correlated with blood parameters in COVID-19 patients. The color and intensity denote the spearman correlation direction and coefficient, where only the significant correlations with |correlation coefficient| > 0.3 were shown. [file 40168_2021_1008_MOESM7_ESM.tif]
